# Age‐specific habitat preference, carrying capacity, and landscape structure determine the response of population spatial variability to fishing‐driven age truncation

**DOI:** 10.1002/ece3.7486

**Published:** 2021-03-31

**Authors:** Hsiao‐Hang Tao, Gaël Dur, Po‐Ju Ke, Sami Souissi, Chih‐hao Hsieh

**Affiliations:** ^1^ Institute of Oceanography National Taiwan University Taipei Taiwan; ^2^ Creative Science Unit (Geosciences) Faculty of Science Shizuoka University Shizuoka Japan; ^3^ Department of Ecology and Evolutionary Biology Princeton University Princeton NJ USA; ^4^ Laboratoire d’Océanologie et de Géosciences UMR 8187 CNRS‐ULille‐ULCO Université de Lille Wimereux France; ^5^ Department of Life Science Institute of Ecology and Evolutionary Biology National Taiwan University Taipei Taiwan; ^6^ National Center for Theoretical Sciences Taipei Taiwan; ^7^ Research Center for Environmental Changes Academia Sinica Taipei Taiwan

**Keywords:** age truncation, age‐specific habitat preference, individual‐based model, size‐selective fishing, spatial variability

## Abstract

1. Understanding the mechanisms underlying spatial variability of exploited fish is critical for the sustainable management of fish stocks. Empirical studies suggest that size‐selective fishing can elevate fish population spatial variability (i.e., more heterogeneous distribution) through age truncation, making the population less resilient to changing environment. However, species differ in how their spatial variability responds to age truncation and the underlying mechanisms remain unclear.
2. We hypothesize that age‐specific habitat preference, together with environmental carrying capacity and landscape structure, determines the response of population spatial variability to fishing‐induced age truncation. To test these hypotheses, we design an individual‐based model of an age‐structured fish population on a two‐dimensional landscape under size‐selective fishing. Individual fish reproduces and survives, and moves between habitats according to age‐specific habitat preference and density‐dependent habitat selection.
3. Population spatial variability elevates with increasing age truncation, and the response is stronger for populations with stronger age‐specific habitat preference. On a gradient landscape, reducing carrying capacity elevates the relative importance of density dependence in habitat selection, which weakens the response of spatial variability to age truncation for populations with strong age‐specific habitat preference. On a fragmented landscape, both populations with strong and weak age‐specific habitat preferences are restricted at local optimal habitats, and reducing carrying capacity weakens the responses of spatial variability to age truncation for both populations.
4. *Synthesis and applications*. We demonstrate that to track and predict the changes in population spatial variability under exploitation, it is essential to consider the interactive effects of age‐specific habitat preference, carrying capacity, and landscape structure. To improve spatial management in fisheries, it is crucial to enhance empirical and theoretical developments in the methodology to quantify age‐specific habitat preference of marine fish, and to understand how climatic change influences carrying capacity and landscape continuity.

## Graphical abstract

Using individual‐based model, we show that the interplay between age‐specific habitat preference, environmental carrying capacity, and landscapes structure determines the sensitivity of spatial variability to fishing‐induced age truncation. These findings provide important implications on spatial management in fisheries.

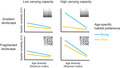

## 1 INTRODUCTION

Populations with highly aggregated spatial distribution are suggested to exhibit less resilience to a changing environment (Hilborn et al., 2003; Hsieh et al., 2008; Kerr et al., 2010; Ruzzante et al., 2006). However, we lack a fundamental understanding of the mechanisms underlying the spatial variability of a population. The importance of this topic has been increasingly recognized in fisheries, as scientists strive to incorporate spatial information of fish populations into management strategies, such as dispersal, landscape‐dependent assemblage, and intraspecific size‐dependent distributions (Bradbury et al., 2008; Ciannelli et al., 2013; Moore et al., 2011; Planque et al., 2011). Thus far for fishes, changes in population size and environmental heterogeneity have been considered as the most critical factors to alter the spatial variability of a population (see review in Hsieh et al., 2010). Recent studies have indicated that another important driver of population spatial variability is its underlying age structure, a property of fish populations that is heavily impacted by age/size‐selective fishing (i.e., age truncation; Charbonneau et al., 2019; Hsieh et al., 2010; Tu et al., 2018). Empirical work has further found that species differ in how their spatial distribution responds to fishing‐induced age truncation (Kuo et al., 2016; Wang et al., 2020), yet the underlying mechanism remains largely unknown.

An essential question is then whether and how the age structure of a population affects its spatial aggregation? A potential answer lies in the fact that conspecific individuals of different ages occupy different habitats. Studies have indicated that individuals exploit various habitats sequentially throughout their life span to maximize fitness at each ontogenetic stage, known as ontogenetic niche shift in habitat use (Werner & Gilliam, 1984; Wilbur, 1980). Such shifts are not only common for holometabolous insects or amphibians but also for marine and freshwater fish and act as a bet‐hedging strategy to maintain population stability (Berkeley et al., 2004; Ryer et al., 2007). Importantly, fish species differ in their degree of age‐specific habitat preference (Winemiller, 1989), a critical life history trait to consider when studying how species’ spatial variability response to fishing‐driven age truncation. The level of age‐specific habitat preference is determined by each age class’ niche width as well as the distance between niche centers (Roos & Persson, 2013). Consider a niche axis (e.g., habitat topology) and a population with strong age‐specific habitat preference. In this case, conspecific age classes have narrow niche widths and long distances between age‐specific niche centers, resulting in a low degree of intrapopulation habitat overlap. Removing old individuals from this population can lead to spatial aggregation and increased spatial variability. In contrast, a population with weak age‐specific habitat preference would have wider niche widths and shorter distances between age‐specific niche centers. This results in highly overlapped habitats between age classes; as such, age truncation may impact less on population spatial variability. While these ideas are conceptually understandable, it is difficult to empirically test the effects of age‐specific habitat preference in isolation. This is because most empirical studies have quantified age‐specific habitat preference based on observed intrapopulation habitat overlaps, a pattern which itself depends on many other factors (Busch & Mehner, 2012; Planque et al., 2011). The lack of accurate information on how different species vary in their age‐specific habitat preferences poses further limitations to associate age‐specific habitat preference with species‐specific responses of spatial variability to fishing.

To gain theoretical understandings of this issue, we aim to develop a model which allows us to simulate different levels of age‐specific habitat preference and to explicitly test how age‐specific habitat preference influences the response of population spatial variability to fishing‐induced age truncation. In addition to age‐specific habitat preference, the response of spatial variability to fishing‐driven age truncation may also depend on the environment's carrying capacity and landscape structure. For example, reducing carrying capacity elevates the relative importance of density‐dependent process in habitat selection, whereas landscape configuration can alter the spatial distribution of fish (Grober‐Dunsmore et al., 2008; Johnson & Heck, 2006; Moore et al., 2011). These alternations potentially influence preference‐dependent processes in fish habitat selection, leading to different population spatial patterns. A recent modeling work has examined changes in spatial variability under fishing mortality and recruitment synchrony for metapopulations (Okamoto et al., 2020), yet it has not investigated how age‐specific habitat preference of fish populations alters population spatial variability. In addition, some studies have suggested age‐specific habitat preference as a potential driver causing elevated population spatial variability under fishing (Ciannelli et al., 2013; Hsieh et al., 2008, 2010; Kuo et al., 2016). However, there is a lack of evidence on the interactive effects of age‐specific habitat preference, carrying capacity, and landscape structure on population spatial variability for exploited fish. This mechanistic understanding of population spatial variability is crucial especially in this era with changing climate, which has predicted to alter the carrying capacity and landscape structure of marine ecosystems (Woodworth‐Jefcoats et al., 2017).

In this study, we examine (a) how population spatial variability changes with fishing‐induced age truncation, (b) how age‐specific habitat preference influences the response of spatial variability to fishing‐induced age truncation, and (c) how carrying capacity and landscape structure alter the influence of age‐specific habitat preference on spatial variability under fishing. To this end, we develop an individual‐based model to simulate the movement, reproduction, and survival processes of individuals of an age‐structured fish population on a two‐dimensional landscape. The fish population has either strong or weak age‐specific habitat preference, and the individual movement is simulated under different carrying capacities and landscape structures. We then study how population spatial variability responds to fishing‐driven age truncation by introducing fishing mortality to elder age classes. We hypothesize that (a) fishing‐induced age truncation elevates spatial variability, (b) age truncation elevates spatial variability more strongly for the fish population with stronger age‐specific habitat preference, and (c) carrying capacity and landscape structure alter fish habitat selection processes, which in turn influence the effects of age‐specific habitat preference on spatial variability under fishing.

## 2 MATERIALS AND METHODS

### 2.1 Model overview

**FIGURE 1:**
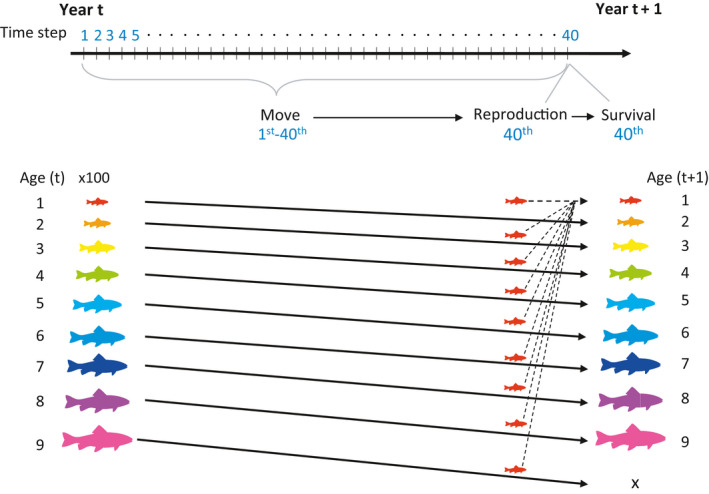
Schematic flowchart of the simulation design. There are 40 time steps in one simulation year. Fish make movement decisions at each time step. At every 40th time step (end of each simulation year), fish reproduce to generate age‐one fish, and the survived fish become one year older. Reproduction and survival processes follow age‐specific reproduction and survival probabilities

We build an individual‐based model to explore mechanisms underlying fish spatial variability under fishing. The model tracks individuals of an age‐structured fish population on a two‐dimensional landscape. The model runs for 15 simulation years, and each year is composed of 40 discrete time steps. The fish population has nine age classes with either strong or weak age‐specific habitat preference. Fish individuals have three processes as follows: movement, reproduction, and survival. Fish make movement decisions at each time step according to a habitat selection function. At the end of each simulation year, fish reproduce and become one year older, based on age‐specific probabilities of reproduction and natural mortality. Six fishing scenarios of different intensities (including a baseline scenario of no fishing) are imposed on elder age classes at the end of each year. The simulations are run on either a gradient or fragmented landscape with six levels of carrying capacities. The parameter combinations include 2 age‐specific habitat preferences × 6 fishing intensities × 2 landscape structure × 6 carrying capacities, thereby generating 144 unique combinations. For each unique combination, we run 60 sets of simulation replicates. In the beginning of the simulation, 900 fish individuals (100 individuals for each age class) are randomly located on the landscape (Table 1). We obtain the local abundance of each age class in each cell for each simulation year, and we calculate age diversity and population spatial variability indices. The individual‐based model is built and run on the Mobidyc platform (Ginot et al., 2002). A schematic flowchart of the simulation is provided in Figure 1. Details of the model design are provided below.

**TABLE 1:** Model scenarios used for testing the different hypotheses

Hypothesis	Parameter	Value	Expected regression slope of spatial variability as a function of age diversity
H1—fishing‐induced age truncation elevates spatial variability	Fishing mortality of age class 4–8 (%)	0%, 10%, 20%, 30%, 40%, 50%	The slope is different from 0
H2—population with strong age‐specific habitat preference has higher spatial variability under age truncation	Distance between niche centers of the youngest and oldest age class	Strong: 0.6 Weak: 0.2	Difference in the slope exists between strong and weak habitat preferences
	Niche width	Strong 0.4 Weak 0.8	
H3a—Carrying capacity alters the effect of age‐specific preference on spatial variability and age diversity relationship	Environmental carrying capacity	5, 10, 20, 40, 80, 160	The difference in the slope between strong and weak habitat preferences varies with carrying capacity
H3b—Landscape structure alters the influence of carrying capacity on the relationship between age‐specific preference, age diversity, and spatial variability	Landscape structure	Gradient, fragmented	The difference in the slope between strong and weak habitat preferences with varying carrying capacity differs between the two landscapes

Note

The number of parameters modified for testing the hypothesis cumulates. For example, for testing hypothesis 2, we run simulations with different fishing mortality rates and different niche parameters. Strong, strong age‐specific habitat preference; Weak, weak age‐specific habitat preference.

### 2.2 Landscape

The two‐dimensional landscape represents the distribution range of the fish population. The landscape has a close‐boundary and consists of 11 × 11 grid cells. Each cell represents a habitat patch in the landscape and is characterized by several attributes (Table S1). To create habitats with different intrinsic qualities, cells are evenly divided into eleven groups. Each group of cells is assigned an intrinsic habitat index (*H*), which are evenly spaced discrete numbers ranging from 0 to 1, that is, 0, 0.1, 0.2, 0.3, …1. The intrinsic habitat index refers to the physical environment, for example, temperature, current, topology, which we assume will not be altered by the fish population (i.e., no density‐dependent feedback dynamics with the population). All the cells in the landscape are also assigned a universal environmental carrying capacity (*K*). Environmental carrying capacity refers to resource availability that has feedback loops with the population, determining density‐dependent dynamics during the habitat selection process (see also Section 2.3). Intrinsic habitat index and environmental carrying capacity are assumed to be independent in our model, that is, carrying capacity does not co‐vary with intrinsic habitat quality. When the local abundance exceeds the environmental carrying capacity of a cell, the realized habitat suitability of this cell decreases (see also Equation (1)).

To examine how landscape structure influences population spatial distribution, the cells are arranged either as (a) a gradient landscape, where there is a smooth change in habitat index across the landscape, or (b) a fragmented landscape, where cells are randomly located across the landscape (Figure S1). Oceanic fragmented landscape refers to highly heterogeneous physical environments in space. In addition, to examine how environmental carrying capacity influences population spatial dynamics, we ran the model under six levels of carrying capacities (*K* = 5, 10, 20, 40, 80, and 160, respectively).

### 2.3 Fish

#### 2.3.1 Age‐specific habitat preference

**FIGURE 2:**
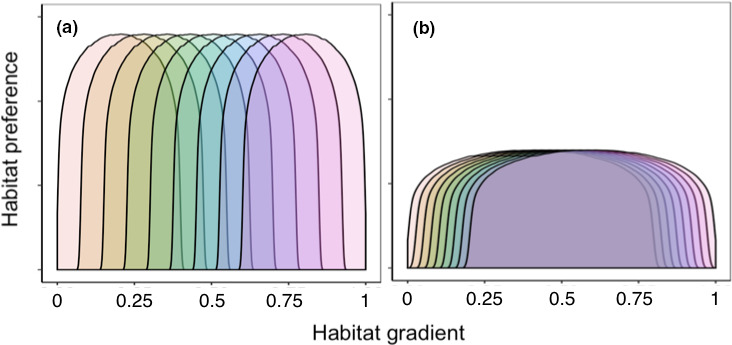
Schematic illustration of a fish population with nine age classes having either strong (a) or weak (b) age‐specific habitat preference, along a habitat range between 0 and 1. Each age class has its niche curve, indicated by a symmetrical beta distribution (alpha = 1.2, beta = 1.2; Table S1). For strong age‐specific habitat preference (a), niche centers of age classes range between 0.2 and 0.8, and the niche width is 0.4. For weak age‐specific habitat preference (b), niche centers of age classes range between 0.4 and 0.6, and the niche width is 0.8

To examine how age‐specific habitat preference influences the relationship between population spatial variability and age truncation, the fish population exhibits either strong age‐specific habitat preference (strong ontogenetic niche differentiation) or weak age‐specific habitat preference (weak ontogenetic niche differentiation). Age‐specific habitat preferences are modeled as niche curves following beta distributions. Within each preference scenario, all age classes have the same symmetrical niche shapes, that is, the means are located at the centroid of the distribution. To factor out the effects of population distribution range on spatial variability, the niche ranges under both scenarios are the same, that is, between 0 and 1. As a consequence, the population spreads over the whole landscape for both preference scenarios. A population with strong age‐specific habitat preference has shorter niche width and longer distance between niche centers of adjacent age classes, resulting in higher age‐specific habitat segregation and thus stronger ontogenetic niche shift. In contrast, a population with weak age‐specific habitat preference has wider niche width and shorter distance between niche centers of adjacent age classes, resulting in highly overlapped habitat preference between age classes and thus weaker ontogenetic niche shift (Figure 2).

At the individual level, each fish is assigned a habitat preference value (*Z*) as its most suitable habitat choice. This value is drawn randomly from the beta distribution corresponding to the age‐specific niche curve that the individual belongs to. The age‐specific habitat preference value of an individual fish is updated when the fish grows older.

#### 2.3.2 Movement

In the beginning of the simulation, fish individuals spread randomly across the landscape. Then, all individuals make movement decisions at each time step (either moving to a neighboring cell, or staying at the original cell) and act synchronously. The movement decision of each individual is based on comparing the current cell's realized habitat suitability value to that of a neighboring cell. If the realized habitat suitability of the neighboring cell is higher than that of the current cell, the location of this fish is updated to this neighboring cell at the next time step. Otherwise, the fish remains at the same location at the next time step.

Realized habitat suitability *R*_(_ *_c_*_,_ *_i_*_)_ of cell *c* for a fish individual of age class *i* is calculated as a habitat selection function modified from the basin model (MacCall, 1990). It is composed of a habitat preference term and a density dependence term:

(1) $$R_{\left(c,i\right)}=1‐\left|H_{c}‐Z_{i}\right|‐\left(N_{c}/K\right)$$

Here, *H_c_* is the intrinsic habitat quality of cell *c*, *Z_i_* is the habitat preference of a fish individual of age class *i*, *N_c_* is the local fish abundance in cell *c*, and *K* is the environmental carrying capacity. The habitat preference term |*H_c_*–*Z_i_*| measures the similarity between the intrinsic habitat quality of a cell and the habitat preference of an individual fish. A higher similarity between the two variables leads to a higher realized habitat suitability. The density dependence term (*N_c_*/*K*) measures the ratio between the local abundance and the environmental carrying capacity. A higher density dependence term leads to a reduced realized habitat suitability due to intra‐population competition.

We add randomness in the choice of moving directions of fish individuals. That is, fish move randomly toward a direction and return to the original place if the new habitat does not provide higher realized suitability (note that in the simulation, the fish simply stays at the same cell at the next time step, rather than moving forward and backward). When translating this behavior into the simulation, a fish individual randomly chooses a neighboring cell among eight candidate neighboring cells, rather than moving directly to the neighbor cell with the highest realized habitat suitability among all. This movement rule is based on recent evidence that fish cannot always to sense the environmental gradient (see review Planque et al., 2011). Therefore, this moving behavior in our simulation follows modified ideal free distribution, which states that individuals are free to move toward a better habitat when there is no restriction in dispersal, nor intra‐ or interspecific competition (Fretwell & Lucas, 1970; Morisita, 1952).

The randomness in the moving direction delays the time steps that a fish needs to reach the optimal cells in the landscape. Without the constraints in carrying capacity and landscape structure, all fish individuals can reach their preferred habitats within 40 time steps. Based on empirical observations, fish populations exhibit stage‐specific distributions, that is, mature fish aggregate at spawning grounds (see review Ciannelli et al., 2014). We therefore assign that one simulation year is composed of 40 time steps, allowing fish to reach their preferred habitats, before turning one year older and pursuing habitats of different intrinsic qualities.

#### 2.3.3 Reproduction, natural mortality, and fishing mortality

The fish population has nine age classes. This setting allows us to generate different levels of age truncation under fishing scenarios while minimizing computational cost. The initial population is composed of 900 individuals (100 individuals for each age class).

Fish individuals reproduce and survive based on the age‐specific reproductive and survival probabilities. Therefore, both reproduction and survival are stochastic processes for fish individuals. The survival rate of newborns is one, thereby the reproductive rate is equivalent to the recruitment rate in our study. For simplicity, the reproduction and survival probabilities are consistent over generations and are independent of habitat quality, carrying capacity, and fishing scenarios.

The reproduction and survival processes occur once a year at the last time step (the 40th step of each year). The newborn age‐one fish individuals are located at the same cells as the mother fish. The reproduction process is followed by the survival process. That is, all fish of age nine die, whereas fish of other ages either die or survive and become one year older. The newborn fish then join the survived individuals from the last simulation year to proceed to the following year. The total population size (*N*) at year *t + *1 can be approximated as follows:

(2) $$N_{t+1}=\sum_{n=1}^{9}\left(1+R_{i}‐M_{i}\right)N_{i,t}‐\sum_{n=4}^{8}F_{i}N_{i,t},$$

where *N_i,t_* is the abundance of age class *i* at year *t*, *R_i_* is the reproductive rate of age class *i*, *M_i_* is the natural mortality rate of age class *i*, and *F_i_* is the fishing mortality of age class *i* (between age 4 and 8).

The age‐specific reproductive and survival rates are assigned using a Leslie matrix, considering population growth rate, age structure, and relative elasticity in population size and age structure.

**FIGURE 3:**
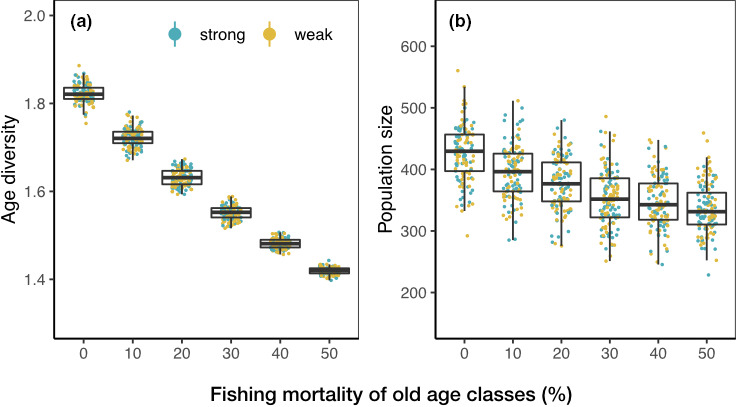
An example of age diversity (a) and population size (b) under different intensities of size‐selective fishing on the gradient landscape with carrying capacity of 40. The simulation is run for a population with either strong (blue) or weak (yellow) age‐specific habitat preference. Each point represents the mean calculated between year 5 and 15. Age diversity is calculated as Shannon index over the landscape. Population size is the total number of total individuals over the landscape. Points are jittered horizontally for visualization. Both age diversity and population size do not differ between strong and weak age‐specific habitat preferences

First, in order to create a population with a nearly constant population size over generations without fishing, we set the population growth rate as 0.999. Second, in order to obtain a right‐skewed age structure for the population as most real‐world fish species, we tune the reproductive and survival rates so that the fish population has a right‐skewed age structure. There are rapid changes in the population size in the first five years, due to the transient dynamics (Figure S2). During this transient period, the number of individuals decreases more with older age classes, leading to right‐skewed demography that is determined by the elements of the eigenvector of the Leslie matrix. After the transient period, the system settles down and the population growth rate reaches a constant value that is determined by the leading eigenvalue of the Leslie matrix. With our designed population growth rate, the population size decreases very little over hundreds of simulation years after the transient period (Figure S2). We therefore use the generated data from the 5th simulation year onward for subsequent analyses. Third, we aim to examine the effects of age diversity on spatial variability while minimizing the effects of population size on spatial variability under size‐selective fishing. We achieve this by assigning relative higher reproductive rates and survival rates to younger age classes than older age classes. Through this design, the newborns generated by abundant younger fish compensate for the abundance reduction of older fish. As a result, changes in age diversity are more pronounced than changes in population size under fishing (Figure 3). Combining the above three practices, the resulting reproductive rates of age class 1 and 2 are 53% and 50%, respectively, while the rest of the age classes are 1%. The natural mortality rate is 10% for age class 1 and 2, 40% for age class 3 to 8, and 100% for age class 9, respectively. We acknowledge that such demographic settings do not match most of the real‐world fishes; however, by doing so, we minimize the effect of population size on spatial variability (see Figure S3 for alternative parameterization).

**FIGURE 4:**
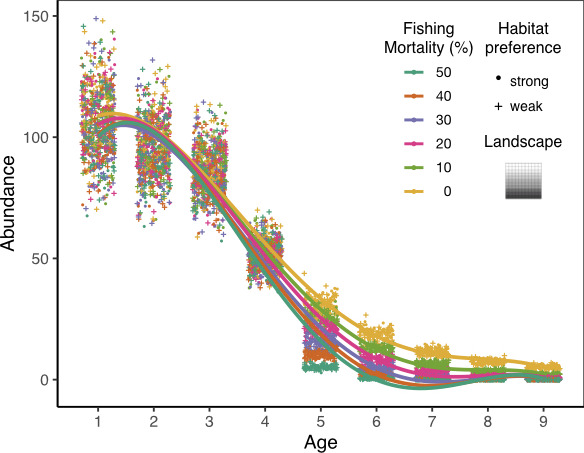
An example of changes in age structure under different intensities of size‐selective fishing on a gradient landscape with carrying capacity of 40. Color indicates different intensities of size‐selective fishing, including a baseline scenario without fishing (fishing mortality = 0). Strong and weak age‐specific habitat preferences are indicated by circles and crosses, respectively. Each point represents the mean abundance of an age class calculated between year 5 and 15. There are 60 simulation replicates for each fishing scenario and habitat preference combinations. *Y*‐axis denotes the abundance of the corresponding age class over the landscape. Points are jittered horizontally for visualization. The age structure is right‐skewed, mimicking most fish species. Age structure becomes more truncated with increasing fishing mortality. There is no difference in the age structure between populations of strong and weak age‐specific habitat preferences

Size‐selective fishing is integrated into the survival process of the population once a year, by adding fishing mortality to exploited age classes, that is, age class 4 to 8. We introduce six fishing intensities by adding fishing mortality of 0%, 10%, 20%, 30%, 40%, and 50% to exploited age classes, respectively. Fishing mortality of 0% is a baseline scenario without fishing. Within each scenario, fishing mortality is the same across exploited age classes and is constant throughout the whole simulation period, assuming constant fishing efforts across age classes and time. The resulting total mortalities (natural mortality plus fishing mortality) are 40%, 50%, 60%, 70%, 80%, and 90% for exploited age classes under six fishing scenarios, respectively. Introducing fishing mortality to exploited age classes results in age truncation and declines in age diversity (Figures 3 and 4).

The population under high fishing intensities has lower population growth rates (i.e., the population growth rate with fishing mortality of 50% is 0.995). This leads to a relatively quicker temporal decline in the population size compared with the baseline scenario of no fishing (Figure S2). In order to minimize the impact of changing population size on population spatial variability while maximizing the simulated time span, we analyze the data output up to 15 simulation years for all fishing scenarios. We note that it is beyond our scope to explore population spatial variability at a low population size, which is likely dominated by stochasticity.

### 2.4 Analyses of model outputs

We extract the simulation outputs from year 5 to 15 for further analyses. For these selected simulation years, we extract data from the last time step (39th time step). At this time step, fish have reached their preferred habitats (without the constraints in carrying capacity and landscape structure), before the onset of the next reproduction and survival round.

At the last time step of each selected year, we extract the total number of individuals in each age class over the landscape and calculate the Shannon index. Here, the Shannon index indicates the age diversity of the whole population over the landscape, with higher values indicating high age diversity and thus weak age truncation. We also extract the total number of individuals (regardless of age class) in each cell to calculate the spatial coefficient of variation (CV). Here, the spatial coefficient of variation represents spatial variability of the population over the landscape, where higher values indicate higher spatial aggregation. For each replicated simulation, we calculate the average Shannon index and spatial CV across the 11 selected years (i.e., year 5 to 15).

To test our hypotheses, we fit univariate linear regression models, including spatial variability as a function of age diversity, under each combination of age‐specific habitat preference, carrying capacity, and landscape structure. We fit the regression lines across all six fishing scenarios (i.e., the regression has 6 × 60 points). The slope coefficients derived from the regressions represent the response of spatial variability to age truncation. We extract the slope coefficients to test our three hypotheses as follows (Table 1). Hypothesis 1: Fishing‐induced age truncation elevates spatial variability. To meet this hypothesis, the *slope would be negative,* regardless of age‐specific habitat preference, carrying capacity, and landscape structure. Hypothesis 2: A population with strong age‐specific habitat preference has higher spatial variability under fishing‐induced age truncation. To meet this hypothesis, *populations with strong age‐specific habitat preference have steeper negative slopes*, regardless of carrying capacity and landscape structure. Hypothesis 3: The effects of age‐specific habitat preference on the response of spatial variability to fishing‐induced age truncation differ with carrying capacity and landscape structure. To meet this hypothesis, *the difference in the slopes between strong and weak habitat preferences would vary with different carrying capacities and different landscapes*. To understand the interactive effects of multiple predictors on spatial variability, we also fit two full models with three‐way interactions between age diversity, age‐specific habitat preference, and carrying capacity on either the gradient or fragmented landscape (Table S2). The statistical analyses are conducted with R 1.3.1093 (R Core Team, 2019).

## 3 RESULTS

**FIGURE 5:**
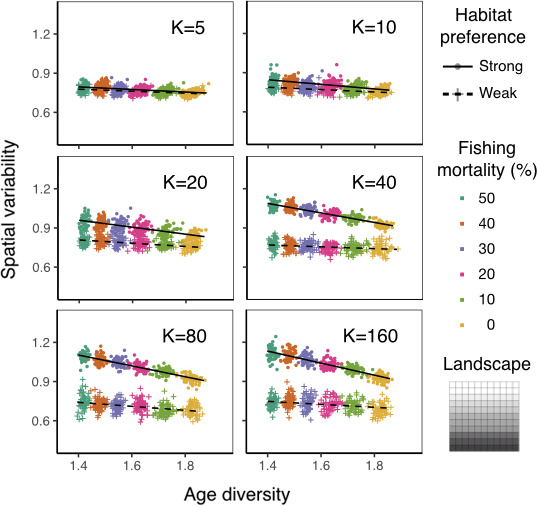
Spatial variability as a function of age diversity at various carrying capacities under strong (circles) or weak (crosses) age‐specific habitat preference on a gradient landscape. Spatial variability is calculated as spatial coefficient of variation (CV) across all habitats over the landscape. Age diversity is calculated as Shannon index over the landscape. Color indicates different intensities of size‐selective fishing. Lines are fitted linear regressions. Each point represents the mean between year 5 and 15 of each simulation replicate

**FIGURE 6:**
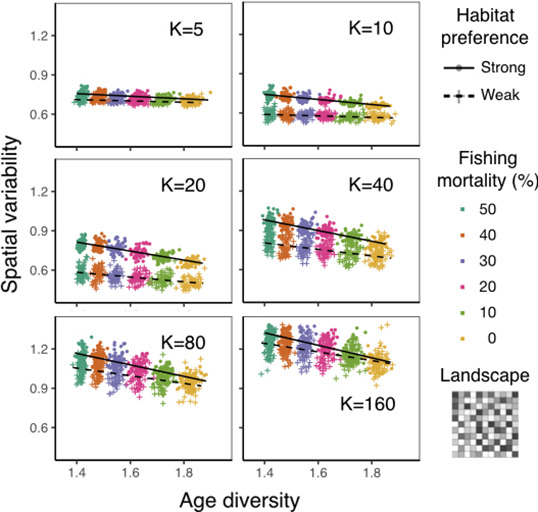
Spatial variability as function of age diversity at various carrying capacities under strong (circles) or weak (crosses) age‐specific habitat preference on a fragmented landscape. Spatial variability is calculated as spatial coefficient of variation (CV) across all habitats over the landscape at each time step. Age diversity is calculated as Shannon index over the landscape at each time step. Color indicates different intensities of size‐selective fishing. Lines are fitted linear regressions. Each point represents the mean between year 5 and 15 of each simulation replicate

**FIGURE 7:**
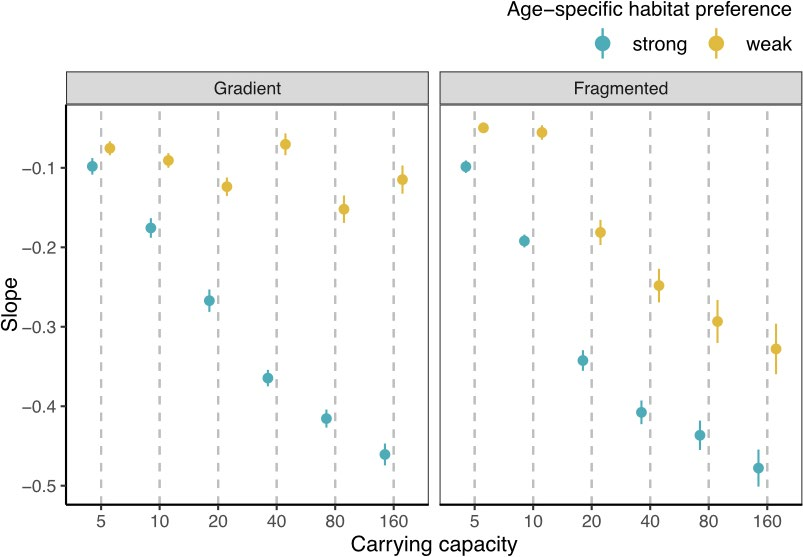
Mean and standard error of the fitted slope coefficient from the linear regression model linking spatial variability (CV) with age diversity (Shannon index), under strong (blue) or weak (yellow) age‐specific habitat preference. The slopes are obtained under various carrying capacities ranging from 5 to 160, on either the gradient or fragmented landscape

The relationships between spatial variability and age diversity (hereafter slope) are always negative, regardless of age‐specific habitat preferences, landscape structure, and carrying capacity (Figures 5, 6, 7 and S4). This finding suggests an increase in spatial variability as size‐selective fishing (Figure 3) decreases age diversity, supporting our H1.

On the gradient landscape, at any given carrying capacity, populations with strong age‐specific habitat preference have more negative slopes compared to the ones with weak age‐specific habitat preference (Figure 5). This implies that age truncation contributes more strongly to spatial variability under strong age‐specific habitat preference compared with weak preference; these findings support our H2. In addition, when carrying capacity decreases, the slope becomes less negative for populations with strong age‐specific habitat preference (Figure 7). That is, decreasing carrying capacity weakens the response of spatial variability to age truncation for populations with strong age‐specific habitat preference. In contrast, for populations with weak age‐specific habitat preference, there is little change in the slope with varying carrying capacity. Together, decreasing carrying capacity reduces the effect of age‐specific habitat preference on the response of spatial variability to age truncation. Statistical analysis also shows a significant three‐way interaction between age diversity, age‐specific habitat preference, and carrying capacity, on spatial variability on the gradient landscape (Table S2).

**FIGURE 8:**
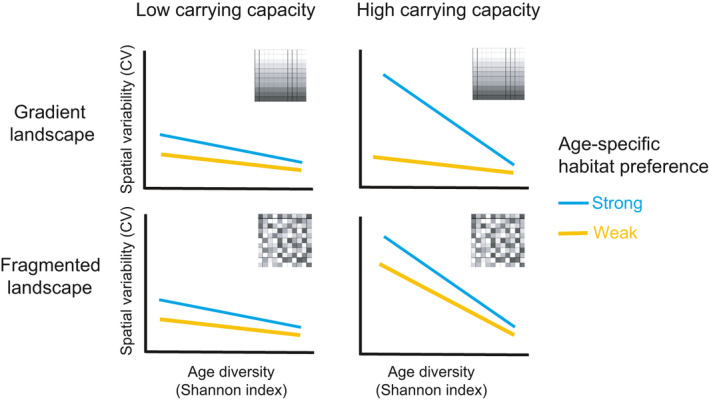
A visual summary of our results showing interactive effects of age‐specific habitat preference, landscape structure, and carrying capacity on the relationship between population spatial variability and age diversity

On the fragmented landscape, under any given carrying capacity, populations with strong age‐specific habitat preference have more negative slopes compared to the ones with weak age‐specific preference, corroborating with the findings on the gradient landscape and supporting our H2 (Figure 6). Interestingly, when carrying capacity decreases, the slope becomes less negative for populations with both strong and weak habitat preferences at similar rates (Figure 7). As such, there is a lack of significant three‐way interaction between age diversity, age‐specific habitat preference, and carrying capacity on spatial variability under the fragmented landscape (Table S2). This result suggests that, under the fragmented landscape, decreasing carrying capacity does not affect the relative influence of strong and weak habitat preferences on the response of spatial variability to age diversity, a result differing from that of the gradient landscape. A visual summary of the results is illustrated in Figure 8.

Population size exhibits negative relationships with spatial variability (Figure S3). In this study, we minimize the changes in population size under fishing to explicitly test the influence of age diversity on spatial variability. Therefore, population size has little confounding effects on the slope between spatial variability and age diversity. Nevertheless, additional simulations with varying population size and age diversity show that the sign of the slopes and the relative differences between strong and weak age‐specific habitat preference remain qualitatively the same as the results of original settings (Figure S3). These evidence additionally support our findings that fishing‐induced age diversity is an important driving factor for population spatial variability.

## 4 DISCUSSION

Our results from the individual‐based model support the first hypothesis that age truncation elevates spatial variability (Figures 5, 6, 7 and S4). This pattern is driven by age‐specific habitat preference, that is, conspecific individuals of different age classes occupy different habitats. When size‐selective fishing removes large individuals from their habitats, there is a sharp decrease in local density in these habitats. In contrast, habitats where younger individuals reside remain highly occupied. Such an increase in variability in local abundances leads to an elevated population spatial variability at the landscape scale. This mechanism may explain empirical findings of the negative association between age structure and spatial variability of fishes (Kuo et al., 2016; Wang et al., 2020).

From the model results, we also observe that relaxation of local density in habitats where old individuals reside leads to an increase in realized habitat suitability, driving some young individuals to these habitats. However, such movement between habitats can be limited when the preference‐dependent process dominates over the density‐dependent process for habitat selection (i.e., when carrying capacity is high, or the population has strong age‐specific habitat preference).

To further demonstrate the role of age‐specific habitat preference on population spatial dynamics, we simulate fish populations with strong and weak age‐specific habitat preferences. Supporting our second hypothesis, the response of spatial variability to age truncation is higher for populations with stronger age‐specific habitat preference, regardless of carrying capacity and landscape structure (Figures 5, 6, 7 and S4). Our simulation shows that populations with strong age‐specific habitat preference have clear spatial segregation between age classes, and the habitat area of each age class is relatively small. In this case, removing older individuals from these populations can greatly increase spatial variability. In contrast, populations with weak age‐specific habitat preference have highly overlapped areas between age classes, and the habitat area of each age class is relatively large. Thus, removing older individuals from these populations only mildly elevates spatial variability. These findings highlight the importance of quantifying and comparing the relative degree of age‐specific habitat preference of fishes to better predict population spatial dynamics. However, only few empirical studies have examined patterns of ontogenetic niche shift for multiple species at the same marine ecosystem (e.g., Winemiller, 1989). In addition, referring age‐specific habitat preference from empirically observed distribution pattern is not always reliable, because observed spatial distribution may be additionally driven by other factors, such as population density, environmental conditions, interspecific interactions, and resource availability (Frank et al., 2018). Systematic assessment and estimation of age‐specific habitat preference among fish species using modeling approaches or field tagging merit further consideration.

To test how carrying capacity and landscape structure alter the influence of age‐specific habitat preference on spatial variability, we further simulate fish moving behavior under varying carrying capacity and landscape structure (Hypothesis 3). Reducing carrying capacity and increasing habitat fragmentation both prevent fish individuals from reaching their preferred habitats. On one hand, reducing carrying capacity elevates the relative importance of density‐dependent over preference‐dependent processes during habitat selection, forcing individuals away from their preferred habitats. Such an effect leads to a larger overlapped habitat area between different age classes. On the other hand, habitat fragmentation prevents individuals from following a smooth gradient to explore the whole landscape and reach globally optimal habitats. Rather, individuals are constrained at locally optimal habitats. This pattern is derived from the setting of simulated fish movement behavior, which follows the assumption that fish is capable of sensing local habitats rather than habitats at long distances. Our results also coincide with empirical work showing the influence of landscape composition and configuration on the fish spatial distribution (Grober‐Dunsmore et al., 2008; Johnson & Heck, 2006; Moore et al., 2011). The altered fish moving behavior due to varying carrying capacity and landscape structure leads to changes in population spatial patterns. On a gradient landscape, reducing carrying capacity greatly weakens the preference‐dependent process in habitat selection for populations with strong age‐specific habitat preference (Figures 5, 7 and S4). On a fragmented landscape, in contrast, the movement of individuals from populations of both strong and weak age‐specific habitat preferences are locally constrained. As both populations perform limited preference‐dependent habitat selection, reducing carrying capacity has similar effects on increasing habitat overlap between age classes. Consequently, the response of spatial variability to age truncation weakens with decreasing carrying capacity at a similar rate for both habitat preference scenarios (Figures 6, 7 and S4).

Given the potential importance of carrying capacity and landscape structure on population spatial variability, future research should look into how climate change influences these properties. A recent study has predicted an increase in epipelagic temperatures and a decline in zooplankton densities across the North Pacific Ocean over the 21st century, which may reduce potential carrying capacity by 20%–50% (Woodworth‐Jefcoats et al., 2017). In the same study, potential redistribution in the physical environment and resource availability has also been predicted to further influence fish distribution. An empirical study by Wang et al. (2020) has found that over 25 years, population spatial variability increases with warming temperature in North Sea plaice, saithe, and Atlantic mackerel, while the relationship is reversed for Atlantic cod. Thus, while changing climate can directly influence fish spatial dynamics by reducing population size, its indirect impacts on carrying capacity and habitat structure and the resulting fish spatial distribution require more attention.

Our model provides a possible explanation for variable empirical findings on the relationships between age truncation and spatial variability. For example, Kuo et al. (2016) have examined correlations between age structure and spatial variability for four exploited fishes in the California Current Ecosystem. They have found negative relationships for Pacific sardine and Pacific chub mackerel at multiple time lags, Pacific hake at time lag of 11 years, and no relationship for bocaccio. Similarly, among eight exploited fish species on the North Sea, only Atlantic cod, plaice, and Atlantic mackerel exhibited negative causal relationships between age diversity and spatial variability of fish (Wang et al., 2020). Our findings from the individual‐based model suggest that within the same marine ecosystem, the variability in species’ spatial responses to age truncation is likely associated with their degree of age‐specific habitat preference. In addition, when comparing between different marine ecosystems, an ecosystem with lower carrying capacity and a more fragmented landscape can exhibit an overall weaker response of spatial variability to age truncation. We suggest that to better predict the response of spatial variability to age truncation of marine fish species, it is critical to take age‐specific habitat preference, carrying capacity, and level of landscape fragmentation into consideration.

While our model results provide useful understandings of the important factors explaining the spatial variability of fish, our model has several limitations that warrant discussion. For simplicity, we assume conspecific individuals of different age classes have symmetrical niche shape and the same width. Empirically, however, older conspecific individuals often have larger niche width (Werner & Gilliam, 1984), which may result in a weaker effect of age truncation on elevating spatial variability. In addition, the reproductive and survival rates of simulated populations are not habitat‐dependent in our model, so that the population spatial dynamics is dominated by habitat selection behavior. Along this line, to disentangle the effects of population size and age structure on spatial variability, we deliberately design age‐specific vital rates in order to maintain relatively unchanged population size under size‐selective fishing. In natural systems, size‐selective fishing influences population size and age structure simultaneously and both properties influence spatial variability. In addition, we did not consider other fish behaviors and traits which potentially determines their movement, such as site fidelity, schooling, memory, avoidance of predation, or age‐specific swimming ability (for a review see Planque et al., 2011). Finally, the carrying capacity is the same among all the habitats while incorporating age‐specific carrying capacity could be closer to real‐world settings. Future models can incorporate various spatial and temporal scales, landscape structures, and age structures to explore species‐ and region‐specific patterns.

## 5 CONCLUSION

Understanding the mechanisms underlying spatial variability of exploited fish is critical for the sustainable management of fish stocks. Empirical studies have shown that fishing‐driven age truncation can elevate the spatial variability of some exploited fishes, yet the underlying mechanisms remain elusive. Using an individual‐based model, we show that the interplay between age‐specific habitat preference, environmental carrying capacity, and landscapes structure determines the sensitivity of spatial variability to age truncation. There are three key findings out of our theoretical investigation. First, age truncation generally elevates spatial variability. Second, the response of spatial variability to age truncation is higher for populations with stronger age‐specific habitat preference, regardless of carrying capacity and landscape structure. Third, carrying capacity and landscape structure alter the fish habitat selection process, which in turn influence the effects of age‐specific habitat preference on spatial variability under fishing. Our findings have important implications for spatial management in fisheries. First, empirical and theoretical developments in the methodology to quantify age‐specific habitat preference of marine fish are important to understand fish spatial dynamics. Second, further investigations on how climatic change influences carrying capacity and landscape continuity are useful to predict fish spatial dynamics under exploitation. Third, our model demonstrates that to track and predict the changes in population spatial variability under exploitation, it is essential to consider the interactive effects of age‐specific habitat preference, carrying capacity, and landscape structure.

## CONFLICT OF INTEREST

The authors declare no conflicting interests.

## AUTHOR CONTRIBUTION

**Hsiao‐Hang Tao:** Conceptualization (lead); Formal analysis (lead); Methodology (lead); Software (lead); Visualization (lead); Writing‐original draft (lead). **Gaël Dur:** Conceptualization (supporting); Formal analysis (supporting); Methodology (lead); Software (supporting); Visualization (supporting); Writing‐review & editing (lead). **Po‐Ju Ke:** Formal analysis (supporting); Visualization (supporting); Writing‐review & editing (lead). **Sami Souissi:** Funding acquisition (supporting); Project administration (supporting); Software (supporting); Writing‐review & editing (supporting). **Chih‐hao Hsieh:** Conceptualization (lead); Funding acquisition (lead); Methodology (supporting); Project administration (lead); Supervision (lead); Writing‐review & editing (supporting).

## Supporting information

Supplementary Material

## Data Availability

Program codes to run all simulations, generate plots, and reproduce statistical analyses in this manuscript are available in the Dryad Digital Repository (https://doi.org/10.5061/dryad.dv41ns1xp).
