# Supplementary material for: Age‐specific habitat preference, carrying capacity, and landscape structure determine the response of population spatial variability to fishing‐driven age truncation
Source: Ecol Evol. 2021 Mar 31;11(11):6358–70. doi: 10.1002/ece3.7486 (PMC8207361; doi:10.1002/ece3.7486)

# Supplementary materials

Table S1. Attribute list of the entities used in the model.

| Attribute | Owner | Dimension | Definition |
| --- | --- | --- | --- |
| *Lc* | Cell | None | ID number of the cell *c* on the gridded landscape |
| *H_c_* | Cell | None | Intrinsic habitat quality index - *H_c_* in Eq(1) (intermediate) |
| *N_c_* | Cell | [ind] | Local density in cell *c* - *N_c_* in Eq (1) |
| *C_i_* | Fish | None | ID number of the cell in which fish *i* is located |
| *α, β* | Fish | None | Shape parameters used to define the shape of the beta distriubtion curve as individual niche curve (intermediate) |
| *Z_i_* | Fish | None | Individual habitat preference value of the fish in age class *i* – *Z_i_* in Eq (1) |
| *S* | Fish | None | Location of the randomly selected surrounding cell *s* |
| *R(c,i)* | Fish | None | Realized habitat suitability for fish of age *i* - *R(c,i)* in Eq (1) – in its current cell *c* |
| *R(s,i)* | Fish | None | Realized habitat suitability for fish of age *i* - *R(s,i)* in Eq (1) - in a randomly selected surrounding cell *s* |
| *D* | Fish | Time step | Number of time steps for each simulation year |

Table S2. Summary statistics of linear regression models from simulation outputs of the continuous landscape (a) and fragmented landscape (b). There are 60 replicates for each combination of age-specific habitat preference, carrying capacity, fishing mortality, and landscape structure. For each simulation replicate, we calculate mean CV and Shannon index by averaging values at the 39^th^ time step of simulation year 5 to 15. Age-specific habitat preference is fitted as a categorical variable with two levels (strong and weak). Age diversity and carrying capacity are fitted as continuous variables. Adjusted R-squared values were 0.78 and 0.87 for continuous and random landscapes, respectively. Significance *** denotes for p-value <0.0001 and ** for p-value < 0.01.

(a) Response variable: spatial variability at continuous landscape

| Predictor | Sum  of  Squares | *df* | Mean  Square | *F* | *p* |
| --- | --- | --- | --- | --- | --- |
| Shannon | 3.333 | 1 | 3.333 | 953.010 | < 2.2e-16*** |
| preference | 33.396 | 1 | 33.396 | 9548.280 | < 2.2e-16*** |
| K | 4.092 | 1 | 4.092 | 1170.060 | < 2.2e-16*** |
| Shannon x preference | 0.755 | 1 | 0.755 | 215.989 | < 2.2e-16*** |
| Shannon x K | 0.320 | 1 | 0.320 | 91.430 | < 2.2e-16*** |
| preference x K | 10.994 | 1 | 10.994 | 3143.446 | < 2.2e-16*** |
| Shannon x preference x K | 0.198 | 1 | 0.198 | 56.576 | 6.542e-14 *** |
| Error | 15.081 | 4312 | 0.003 |  |  |

*Shannon: age diversity; preference: age-specific habitat preference; K: carrying capacity.*

(b) Response variable: spatial variability at fragmented landscape

| Predictor | Sum  of  Squares | *df* | Mean  Square | *F* | *p* |
| --- | --- | --- | --- | --- | --- |
| Shannon | 5.574 | 1 | 5.574 | 839.274 | < 2.2e-16*** |
| preference | 12.037 | 1 | 12.037 | 1812.211 | < 2.2e-16*** |
| K | 173.161 | 1 | 173.161 | 26070.660 | < 2.2e-16*** |
| Shannon x preference | 0.376 | 1 | 0.376 | 56.665 | 6.255e-14 *** |
| Shannon x K | 0.831 | 1 | 0.831 | 125.172 | < 2.2e-16 *** |
| preference x K | 0.585 | 1 | 0.585 | 88.142 | < 2.2e-16 *** |
| Shannon x preference x K | 0.004 | 1 | 0.004 | 0.659 | 0.4169 |
| Error | 28.640 | 4312 | 0.007 |  |  |

*Shannon: age diversity; preference: age-specific habitat preference; K: carrying capacity.*

Figure S1. Different arrangements of the two-dimensional landscapes tested with the model: continuous landscape (a) and fragmented landscape (b). The grey scale colors represent the intrinsic habitat index values between 0 and 1.

**Figure S2** Temporal changes in the population size under the baseline scenario without fishing (a) and the intensive fishing scenario of 50% fishing mortality (b), generated by the corresponding Leslie matrices. For the baseline scenario, the population growth rate is 0.999. After removing stochasticity in reproduction and mortality, the initial population size of 900 declines to 436 after 5 simulation years, and then declines slowly for the following hundreds of simulation years. For example, at simulation year 200, the population size is 406 (a). In contrast, under the most intensive fishing scenario, the population growth rate is 0.995. The initial population size of 900 decreases to 343 after 5 simulation years and reaches 146 at year 200. Compared to the baseline scenario, the decline in the population size is quicker under the intensive fishing scenario (b). Two vertical lines in each plot indicate the population size at simulation year 5 and 15. We extract the simulation data within this period to analyze population spatial variability.

Figure S3. Simulation results from the original setting with variable age diversity and small population size range (a, c, e), and additional setting with variable age diversity and variable population size (b, d, f).


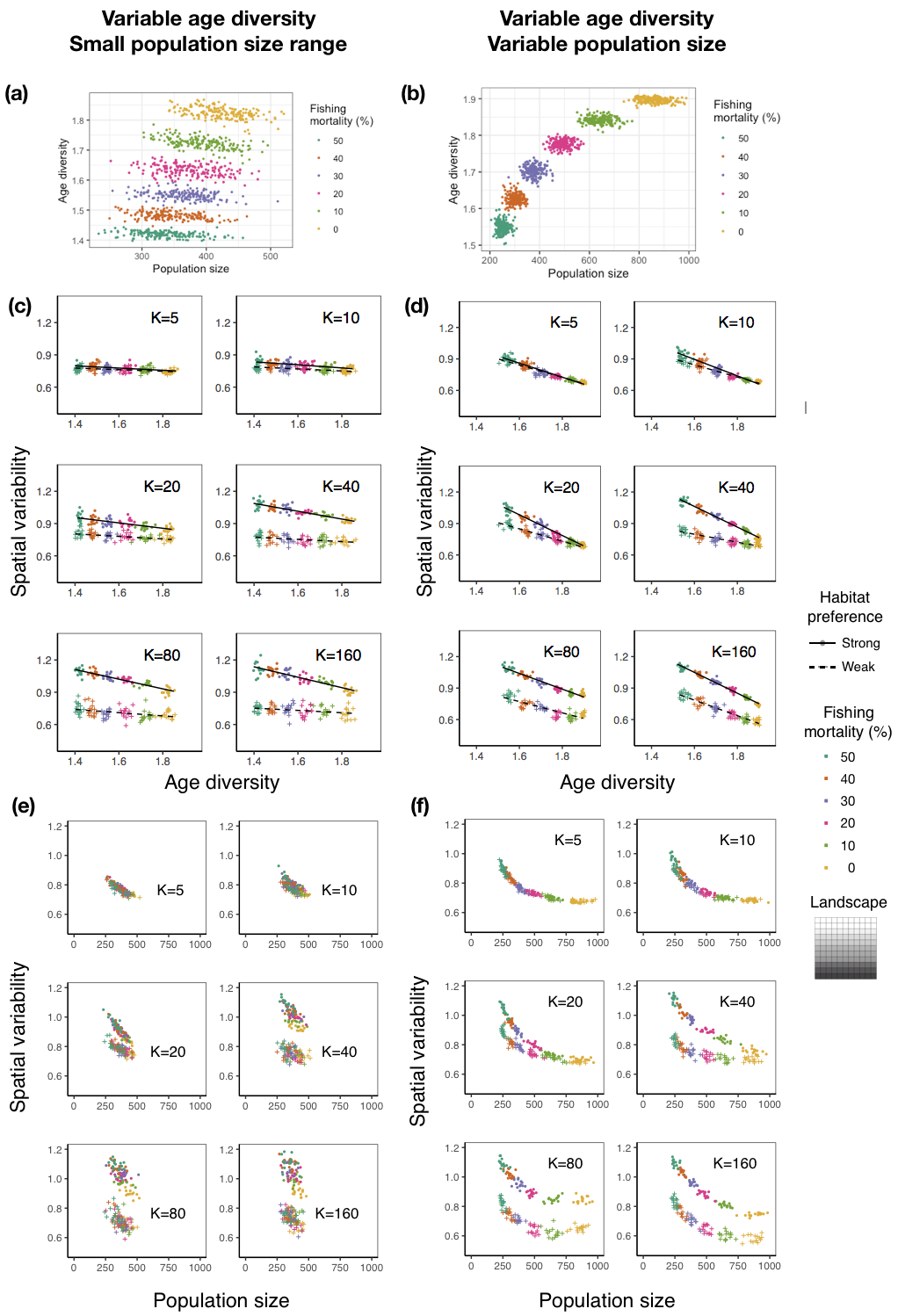


Additional note for Figure S3.

In this additional simulation (panel b, d, f), reproduction rates for age 1 to 9 are 0.1, 0.4, 0.5, 0.5, 0.5, 0.5, 0.5, 0.5, 0.5, respectively. Survival rates for age 1 to 9 are 0.5, 0.7, 0.8, 0.8, 0.8, 0.8, 0.8, 0.8, 0, respectively. We introduce the same fishing mortality scenarios from 0 to 0.5 to fish individuals of age 4 to 8. We run the model on the gradient landscape for 15 replicates. The simulation years and time steps are the same as the original setting. For the comparison purpose, we subset 15 replicates out of 60 replicates from the original simulation and plot the same graphs (panel a, c, e).

In the original setting, population size is controlled within a small range among different fishing scenarios, and age diversity is largely independent of population size (a). The slope between spatial variability and age diversity thus indicates that the slope between spatial variability and age diversity is not confounded by population size (c). Similarly, the slope between spatial variability and population size indicates that the slope between spatial variability and population size is not confounded by age diversity.

In comparison, in the new setting, population size decreases with higher intensity of fishing, and there is a positive relationship between population size and age diversity (b). The slope between spatial variability and age diversity (d) reflects that the relationship between spatial variability and age diversity can be confounded by population size, due to their strong positive correlation. This is supported by the fact that the slopes are more negative in the new setting compared to the original setting (c) under the same age-specific habitat preference and carrying capacity. Similarly, the slope between spatial variability and population size (f) reflects the collective influence of age diversity and population size on spatial variability.

The new simulation setting of varying population size and age diversity does not influence the sign of the slopes, or the differences between strong and weak age-specific habitat preferences (d), compared to the original setting (c). This result therefore supports our conclusion that fishing-induced age diversity is a driving factor for population spatial variability.

Figure S4. Slope coefficients of linear regression linking spatial variability with age diversity under strong (blue) and weak (yellow) age-specific habitat preferences with different carrying capacities (K) and landscape structures. The coefficients are obtained under various carrying capacities ranging from 5 to 160 on either gradient (denoted as grad) or fragmented landscapes (denoted as frag). Averaged slopes of linear regressions linking spatial variability with age diversity are shown as dots, and standard errors of 60 simulation replicates are shown as vertical lines.


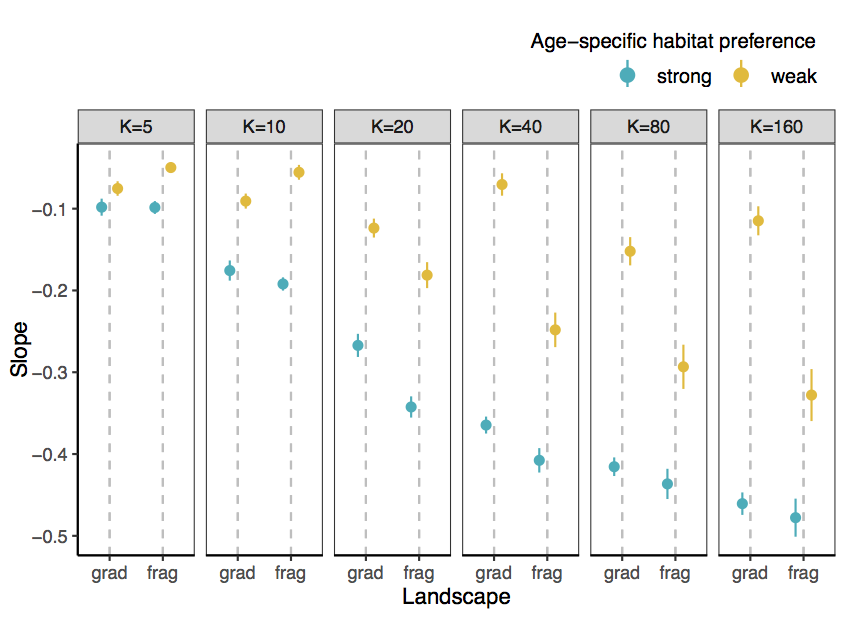

Supplement: Supplementary file 1 — Supplementary Material [file ECE3-11-6358-s001.docx]
